# A medical multimodal large language model for future pandemics

**DOI:** 10.1038/s41746-023-00952-2

**Published:** 2023-12-02

**Authors:** Fenglin Liu, Tingting Zhu, Xian Wu, Bang Yang, Chenyu You, Chenyang Wang, Lei Lu, Zhangdaihong Liu, Yefeng Zheng, Xu Sun, Yang Yang, Lei Clifton, David A. Clifton

**Affiliations:** 1 Institute of Biomedical Engineering, Department of Engineering Science, University of Oxford, Oxford, UK; 2 Jarvis Research Center, Tencent YouTu Lab, Beijing, China; 3 School of Computer Science, Peking University, Beijing, China; 4 Yale University, New Haven, CT USA; 5 Oxford-Suzhou Centre for Advanced Research, Suzhou, China; 6 School of Public Health, Shanghai Jiao Tong University School of Medicine, Shanghai, China; 7 Nuffield Department of Population Health, University of Oxford, Oxford, UK

**Keywords:** Health care, Biomedical engineering

## Abstract

Deep neural networks have been integrated into the whole clinical decision procedure which can improve the efficiency of diagnosis and alleviate the heavy workload of physicians. Since most neural networks are supervised, their performance heavily depends on the volume and quality of available labels. However, few such labels exist for rare diseases (e.g., new pandemics). Here we report a medical multimodal large language model (Med-MLLM) for radiograph representation learning, which can learn broad medical knowledge (e.g., image understanding, text semantics, and clinical phenotypes) from unlabelled data. As a result, when encountering a rare disease, our Med-MLLM can be rapidly deployed and easily adapted to them with limited labels. Furthermore, our model supports medical data across visual modality (e.g., chest X-ray and CT) and textual modality (e.g., medical report and free-text clinical note); therefore, it can be used for clinical tasks that involve both visual and textual data. We demonstrate the effectiveness of our Med-MLLM by showing how it would perform using the COVID-19 pandemic “in replay”. In the retrospective setting, we test the model on the early COVID-19 datasets; and in the prospective setting, we test the model on the new variant COVID-19-Omicron. The experiments are conducted on 1) three kinds of input data; 2) three kinds of downstream tasks, including disease reporting, diagnosis, and prognosis; 3) five COVID-19 datasets; and 4) three different languages, including English, Chinese, and Spanish. All experiments show that our model can make accurate and robust COVID-19 decision-support with little labelled data.

## Introduction

Recently, the rapid development of deep neural networks has enabled their wide applications in clinics^1,2^. To process clinical data of different modalities, different neural networks have been employed accordingly. For processing visual data such as dermoscopy images, Convolutional Neural Network (CNN) based frameworks^3^ have been applied to classify the type of skin lesion^4^; For textual input such as Electronic Medical Record (EMR), Transformer based frameworks^5^ have been be applied to estimate the mortality or re-hospitalisation probabilities^6^; For multi-modal data such as radiology image-report pairs, the encoder-decoder based frameworks^7–11^ have been applied to generate textual reports from medical images.

Deep neural networks can assist physicians in the diagnosis process and relieve their heavy burden. Most deep neural networks exploit supervised training, and therefore their performance heavily relies on the volume and quality of labelled data. However, the labelling process of clinical data is usually costly and time-consuming. For rare diseases, it is difficult to collect and label sufficient data in a timely manner to train a deep learning model (with some studies taking over one year to collect sufficient data^12,13^), thus delaying the rapid deployment of deep learning models needed for combating rare diseases promptly.

Take the recent pandemic SARS-CoV-2/COVID-19 for example, which not only leads to multi-organ failures and death but also threatens to affect global health for the foreseeable future^14^. Although early COVID-19 incurred a high mortality rate, its most recent variants are not life-threatening for the young healthy population. It is still uncertain whether a new variant in the future would pose a life-threatening risk again. Considering the large volume of the vulnerable population for COVID-19, three common types of AI-based decision-support tools can be developed to support accurate diagnosis and prognosis:

- COVID-19 radiology reporting: Given radiology images, physicians need to write textual reports to address the clinical findings^7,11,15–17^. Given the large number of COVID-19 patients, writing medical reports is a heavy burden for physicians who could otherwise concentrate on patient care^18,19^. The overly-heavy workload of physicians is well-documented^20,21^, and using deep learning methods to automatically generate reports that can be modified and approved by physicians can partly automate routine tasks^1,2,22,23^.
- COVID-19 diagnosis: Currently, the Reverse Transcription Polymerase Chain Reaction (RT-PCR) is recognised as the gold standard for COVID-19 diagnosis^24^. Due to the high false-negative rate of RT-PCR and shortage of equipment^25,26^, different diagnosis models that use medical data across different modalities^1,27^ to generate more timely results than RT-PCR can work as an alternative in COVID-19 diagnosis.
- COVID-19 prognosis: A prognosis model^2^ can support better triage on who to admit to the hospital or intensive care, who to isolate, predicting whom and when to recover, and who is at the highest risk of deterioration.

**Fig. 1:**
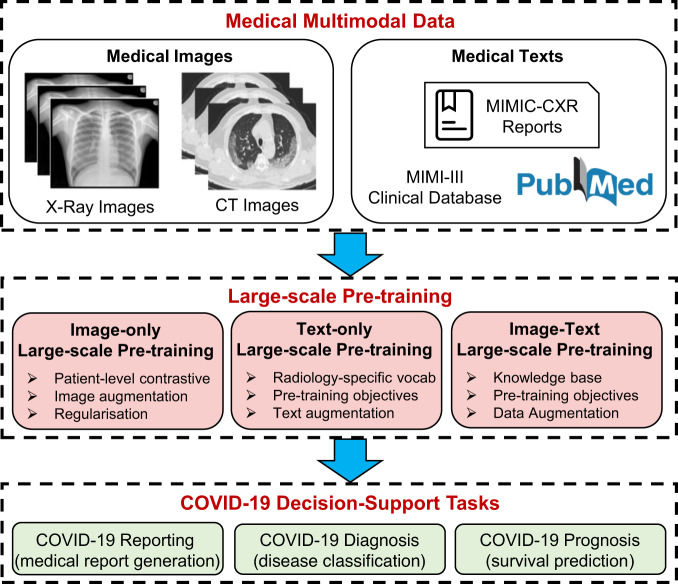
Flowchart. Our presented medical multimodal large language model (Med-MLLM) for COVID-19 reporting, diagnosis and prognosis.

Training common neural networks for the above three tasks requires labels on visual, textual and multi-modal data. However, collecting labelled data for a rare disease is expensive and time-consuming. To this end, inspired by the great success of large-scale pre-training^28–31^, as shown in Fig. 1, we present the *Medical Multimodal Large Language Model* (Med-MLLM) framework for radiograph representation learning^31–34^. Our framework deals with the situation where labelled data are scarce, and shortens the time-frame of model deployment, allowing rapid response to rare diseases in the future.

**Fig. 2:**
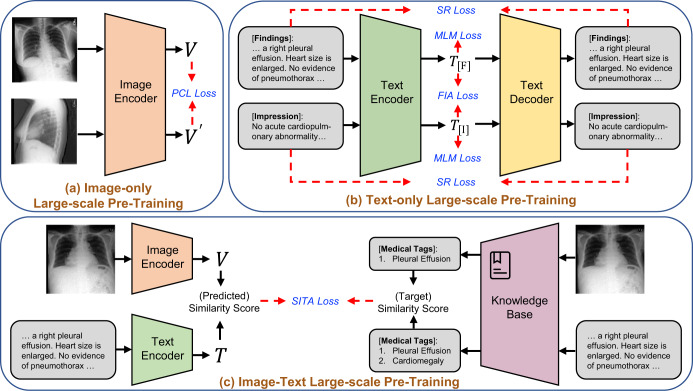
Structure of the presented Med-MLLM framework. It consists of three main components: **a** Image-only pre-training which incorporates the patient-level contrastive learning (PCL); **b** Text-only pre-training which incorporates three training objectives: the masked language modelling (MLM), the sentence reconstruction (SR) loss, and the findings-impression alignment (FIA) loss; and **c** Image-text pre-training which incorporates a knowledge base and a pre-training objective: soft image-text alignment (SITA).

As shown in Fig. 2, our framework adopts multimodal medical data across visual and textual modalities to learn the following comprehensive thorax knowledge. 1) Visual data: for medical images such as Chest X-rays (CXR) and Computed Tomography (CT), we pre-train an image encoder with two types of losses: patient-level contrastive learning loss and image-level contrastive loss. 2) Textual data: for medical texts such as medical reports and clinical notes, we pre-train a text encoder with three types of losses: masked language modelling loss, sentence reconstruction loss, and findings-impression alignment loss. 3) Multi-modal data: for unpaired radiology images and reports, we introduce a soft image-text alignment loss to further pre-train the visual encoder and text encoder. In this manner, Med-MLLM handles visual, textual and multi-modal input, and therefore can be applied to COVID-19 reporting (i.e., medical report generation), diagnosis (i.e., disease classification), and prognosis (i.e., survival prediction) tasks with limited labels for training^1,2,12,13,15,27,35^.

The retrospective and prospective experiments across different modalities, languages, and regions assess the effectiveness of our Med-MLLM for clinical decision-making when using limited labelled data. Besides COVID-19, the framework can be readily applied to other 14 common thorax diseases and tuberculosis as well with 1% labelled data, demonstrating the scalability of our framework in assisting physicians when encountering a rare disease.

Overall, the contributions of our work are as follows:

- With the goal of quick deployment of tools for rapid response to rare diseases, we present the medical multimodal large language model (Med-MLLM) framework. We evaluate the effectiveness of Med-MLLM using the COVID-19 pandemic “in replay”, showing that Med-MLLM is able to accomplish accurate COVID-19 decision-support tasks with limited labelled data. In contrast, existing efforts usually require thousands, or even more, labelled data to achieve similar performance.
- Med-MLLM is able to handle image-only, text-only, and image-text data, addressing multiple medical tasks including reporting, diagnosis, and prognosis. To demonstrate the effectiveness of Med-MLLM, we conduct both retrospective and prospective (i.e., pre-training model from the early COVID-19 and making a prediction for COVID-19-Omicron) experiments across different modalities, languages, and regions.
- To evaluate the scalability of Med-MLLM, we investigate other 14 common thorax diseases and tuberculosis. Our results show that Med-MLLM achieves competitive performances w.r.t. previous works with 1% of the labelled training data, and comparable performance when the full training set is used.

## Overall framework

As shown in Fig. 1, we develop a *Medical Multimodal Large Language Model* (Med-MLLM) for rare diseases to deal with the situation where the labelled data is scarce. An example is the early stages of a new pandemic, for which we will have very little data. Med-MLLM (i) adopts the unlabelled medical image data from existing public image datasets, e.g., chest radiology images^36,37^, COVID chest X-ray images^38–42^, and COVID CT images^40,42–44^ to perform image-only pre-training^45,46^ to learn visual characteristics, capturing the rich diagnostic information in medical images^1,2,27^; (ii) adopts the unlabelled medical text data from existing public text datasets, e.g., PubMed^47^, MIMIC-CXR medical reports^37^, and MIMIC-III clinical notes^48^, to perform text-only pre-training^49–51^ to learn text semantics and clinical findings in medical texts^52^; (iii) adopts an existing large knowledge base, i.e., Unified Medical Language System (UMLS)^53^, to perform image-text pre-training^54^ to unify the learned knowledge from unpaired images and texts, capturing accurate disease phenotypes and clinical presentations.

Figure 2 shows the detailed structure of the Med-MLLM framework. For a fair comparison, we adopt the ResNet-50^55^ as the image encoder and the Transformer^5^ as the text encoder/decoder. In detail, Med-MLLM (i) adopts contrastive learning^46,56^ to perform image-only pre-training, which is improved by a patient-level contrastive learning, image augmentation, and regularisation; (ii) builds a large language model (LLM)^49^, which adopts self-supervised learning^49,50^, to perform text-only pre-training. The LLM is further improved by the radiology-specific vocabulary, two pre-training objectives, and a text augmentation method; (iii) adopts contrastive learning^54^ to perform image-text pre-training, improved by the UMLS knowledge base^53^ and a pre-training objective. In this way, our framework could capture comprehensive medical knowledge to provide a solid basis for the diagnosis of rare diseases, including COVID-19 and its variant–Omicron. As a result, our framework can be taken as a “warm start" algorithm to provide an accurate and efficient diagnosis of rare diseases using limited labels. Our extensive experiments show that the framework yields encouraging performance for a wide range of downstream tasks.

### Fine-tuning

**Fig. 3:**
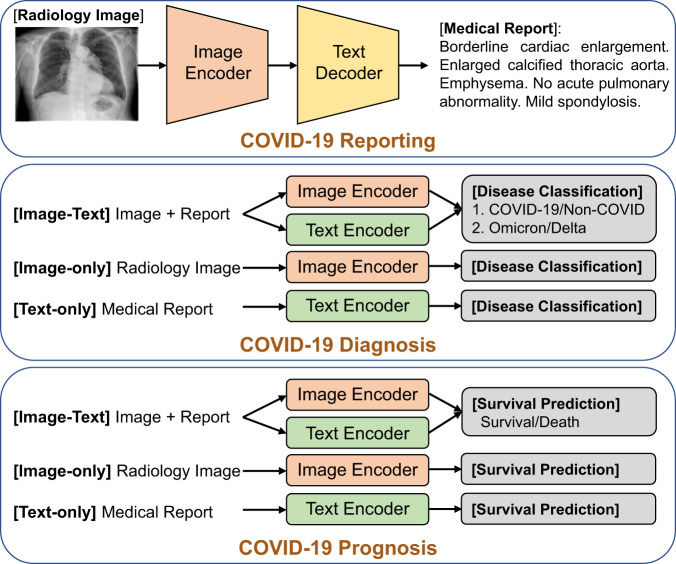
Illustration of fine-tuning our Med-MLLM on downstream COVID-19 decision-support tasks: COVID-19 reporting, diagnosis, and prognosis.

Figure 3 illustrates the details of fine-tuning the Med-MLLM for downstream COVID-19 decision-support tasks. (i) We adopt the image encoder and an additional text decoder to fine-tune (cross-entropy optimisation) the pre-trained Med-MLLM on the COVID-19 reporting (medical report generation) task. (ii) For the task of COVID-19 diagnosis (disease classification), we add a classification layer on the output of image and/or text encoders, and the Med-MLLM is fine-tuned using a binary cross-entropy loss. (iii) For the task of COVID-19 prognosis (survival prediction), we adopt the same fine-tuning strategy as the COVID-19 diagnosis task above, because these two tasks differ solely in the output results. Both tasks can accept three types of input medical data: image-only, text-only, and image-text.

## Results

In this section, we conduct experiments on COVID-19 reporting, diagnosis, and prognosis tasks. We first describe five COVID-19 datasets used for the experiments. Then, we present the results of our framework on COVID-19 decision-support across modalities, languages, and regions, using limited labels (e.g. 1% labelled data).

### Datasets

We evaluate the performance of our framework on five COVID-19 datasets across different modalities, languages, and regions, i.e., COVIDx-CXR-2 dataset^40^, COVID-CXR dataset^39,41^, COVID-19 CT dataset^44^, BIMCV-COVID-19 dataset^42^, and COVID-HCH dataset^16^. The COVIDx-CXR-2 dataset includes 29,986 medical images of 16,648 patients from 51 countries; The COVID-CXR dataset contains over 900 chest X-rays of 412 patients from 26 countries, where 361 patients have survival/death labels. The COVID-19 CT dataset contains 1104 medical images associated with 368 medical reports in Chinese from 96 patients. The dataset was collected from the First Affiliated Hospital of Jinan University Guangzhou and the Fifth Affiliated Hospital of Sun Yat-sen University, Zhuhai, China. The BIMCV-COVID-19 dataset is a large dataset consisting of over 20k CXR and CT images from over 1000 COVID-19 patients along with their radiographic reports in Spanish. The COVID-HCH dataset includes 5115 COVID-19 records and 4112 non-COVID-19 records of viral and bacterial pneumonia from 91 patients, resulting in a total of 9227 records associated with radiographic reports in Chinese. Specifically, the 5115 COVID-19 records are composed of 3577 COVID-19-Delta records and 1538 COVID-19-Omicron records. Meanwhile, we invite clinical professionals to translate 100 reports into English. Each English report is associated with multiple (> 10) medical images, and different images serve as different samples. We adopt the Omicron data to perform simulated prospective studies. In detail, we pre-train the model on Delta data and fine-tune the model on Omicron data.

To pre-process the datasets, we randomly split them into training, validation and test sets with a ratio of 8:1:1, respectively. The training, validation, and test sets are used to train the model, select the optimal modules and hyper-parameters, and evaluate the performance, respectively. All protected health information (e.g., patient name and date of birth) was de-identified for all datasets used in our experiments. Several previous works^57–60^ construct a balanced test set to minimise the effect of dataset bias on model performance. The reason is that a balanced test set provides a genuine reflection of the models’ ability to correctly distinguish between positive and negative cases, i.e., their capability to accurately identify COVID-19 cases. Thus, the models are prevented from exploiting biases in the data distribution to achieve high overall performance. To this end, we constructed balanced validation and test sets by randomly sampling 10% of the dataset, with 5% from the positive cases and the other 5% from the negative cases (i.e. the ratio of COVID-19 records to non-COVID records is 1:1). The remaining 80% samples are used as the training set. Therefore, our models are trained on the unbalanced set, but validated and tested on the balanced set. For all experiments, we conduct multiple runs with different seeds and report the average performances for baselines and our model.

### Experimental settings

In our work, we conduct both prospective and retrospective studies. In the retrospective studies, we perform the experiments by directly pre-training and evaluating the model on the COVID-19 data. For the prospective studies, we perform the experiments by pre-training the model from early COVID-19 and making predictions for COVID-19-Omicron. For example, we have observed the Delta variant but have no data for Omicron, so our prospective studies can test Med-MLLM to see how it adapts to the new variant (i.e., Omicron) from the old variant (i.e., Delta).

### COVID-19 reporting

Our COVID-19 reporting task aims to automatically generate a comprehensive and coherent medical report of a given medical image. In clinical practice, writing reports for numerous images from routine imaging exams can be time-consuming and tedious for even experienced radiologists^7^. Given the large volume of medical images, automatically generating reports can improve current clinical practice in diagnostic radiology and assist radiologists in clinical decision-making. Therefore, automatic report generation is receiving remarkable attention in both communities of artificial intelligence and clinical medicine^7,11,15,16,61–64^. To measure the performance of COVID-19 reporting, we select the widely-used natural language generation metrics, including BLEU-2, -3, -4^65^, ROUGE-L^66^, and CIDEr^67^, which are computed by a standard evaluation toolkit^68^ automatically. These metrics measure the match between the generated reports and reference reports annotated by professional physicians.

#### Retrospective studies

We further select existing methods, including R2Gen^61^, KGAE^62^, and XProNet^63^, for comparison. We conduct retrospective studies on the COVID-19-CT dataset in Chinese and the BIMCV-COVID-19 dataset in Spanish. We randomly select 1% labelled data for training. The results in Table 1 show that with 1% of training data, our method achieves competitive performance w.r.t. the previous models trained on the full training set across Chinese and Spanish. It shows that our approach can be efficiently trained and deployed with limited labels to combat rare diseases promptly. Using the full training set as used in previous methods, our method achieves the best results across different languages and regions. In detail, our framework outperforms previous best results by up to 4.3%/3.8% in BLEU-4, 9.1%/4.3% in ROUGE-L, and 10.9%/9.8% in CIDEr scores in Chinese/Spanish scenarios. The improvement demonstrates the effectiveness of our framework in providing a solid basis for COVID-19 reporting.

**Table 1:** Results of the COVID-19 reporting task: an image-text multimodal task aiming to automatically generate the medical reports of given medical images, on three datasets across Chinese, Spanish and English.

Methods	Year	Ratio of training data	Retrospective studies									
			Dataset: COVID-19-CT (Chinese)					Dataset: BIMCV-COVID-19 (Spanish)				
			BLEU-2	BLEU-3	BLEU-4	ROUGE-L	CIDEr	BLEU-2	BLEU-3	BLEU-4	ROUGE-L	CIDEr
R2Gen^61^	2020	1%	35.9	33.2	31.3	41.7	57.6	28.8	24.3	21.0	37.4	42.7
KGAE^62^	2021	1%	43.6	39.1	36.7	50.2	72.4	35.5	29.8	27.8	45.4	58.0
XProNet^63^	2022	1%	38.4	35.0	33.5	44.8	60.7	32.2	27.0	25.1	40.6	51.6
Med-MLLM	Ours	1%	54.3_(2.7)_	47.5_(2.1)_	42.1_(1.7)_	57.2_(1.9)_	85.3_(2.5)_	47.6_(3.0)_	42.0_(2.3)_	38.1_(1.8)_	55.4_(1.6)_	73.4_(2.8)_
R2Gen^61^	2020	100%	53.3	45.1	39.4	54.5	80.4	43.2	37.8	33.2	52.9	67.2
KGAE^62^	2021	100%	56.4	48.6	44.3	60.3	83.7	47.0	40.6	36.8	53.2	71.3
XProNet^63^	2022	100%	57.7	49.0	44.4	59.4	84.5	48.3	41.1	38.4	54.0	70.9
Med-MLLM	Ours	100%	**64.2**_(2.1)_	**55.0**_(1.6)_	**48.7**_(1.3)_	**68.5**_(1.2)_	**95.4**_(2.0)_	**55.6**_(2.4)_	**46.4**_(1.8)_	**42.2**_(1.4)_	**58.3**_(1.2)_	**80.7**_(2.3)_

Methods	Year	Ratio of training data	Prospective studies									
			Dataset: COVID-19-Omicron (Chinese)					Dataset: COVID-19-Omicron (English)				
			BLEU-2	BLEU-3	BLEU-4	ROUGE-L	CIDEr	BLEU-2	BLEU-3	BLEU-4	ROUGE-L	CIDEr
R2Gen^61^	2020	1%	57.0	52.9	49.2	55.7	74.8	20.3	18.5	14.2	30.7	35.6
KGAE^62^	2021	1%	62.9	56.0	51.3	58.6	83.4	25.7	22.4	18.8	34.6	41.1
XProNet^63^	2022	1%	63.0	57.2	52.2	58.8	84.1	26.0	21.9	18.7	35.0	42.8
Med-MLLM	Ours	1%	70.1_(1.7)_	64.6_(1.4)_	60.1_(1.0)_	64.3_(1.5)_	95.2_(1.1)_	32.4_(2.6)_	28.7_(1.7)_	24.9_(1.5)_	46.7_(2.1)_	53.0_(1.9)_
R2Gen^61^	2020	100%	63.1	57.5	52.1	59.2	85.3	31.7	26.2	20.2	43.1	47.8
KGAE^62^	2021	100%	67.3	58.7	53.0	60.4	89.2	34.2	27.5	24.4	46.0	50.3
XProNet^63^	2022	100%	66.5	59.2	53.3	61.1	90.4	33.0	28.0	24.8	47.8	51.9
Med-MLLM	Ours	100%	**74.5**_(1.4)_	**67.8**_(1.0)_	**63.2**_(0.9)_	**67.2**_(0.8)_	**97.5**_(0.5)_	**40.1**_(1.7)_	**34.4**_(1.2)_	**29.0**_(1.0)_	**51.6**_(1.3)_	**62.6**_(1.4)_

We report the mean and standard deviation_(STD)_ of performance. Higher is better for all metrics. The best results are in bold. 100% denotes that the models are trained on the full training set.

#### Prospective studies

We perform prospective studies on the COVID-19-Omicron data from the COVID-HCH dataset. Specifically, we adopt the Delta data for pre-training the model and adopt the Omicron data for evaluation. As shown in Table 1, our method Med-MLLM outperforms previous methods trained on full training data on most metrics. Compared with retrospective studies, our method achieves better results on COVID-19-Omicron reporting. The results of prospective studies evaluated on COVID-19-Omicron data show that our method shortens the time for data acquisition, allowing us to respond quickly in future to rare diseases across different languages and regions. We further validate it on the following COVID-19 diagnosis and prognosis tasks. It is worth noting that the performance of our method can be further improved by using more training data, achieving improved performances when it is trained with the full training set as used in previous methods.

### COVID-19 diagnosis

In the retrospective setting, the COVID-19 diagnosis task (i.e., disease classification) aims to distinguish COVID-19 from non-COVID-19 cases. In the prospective setting, the aim is to identify COVID-19-Omicron. We conduct retrospective studies on the COVIDx-CXR-2 and COVID-19-Delta data and conduct prospective studies on the COVID-19-Omicron data. In our experiments, we report the widely-used AUC for assessing the diagnosis accuracy.

#### Retrospective studies

We utilise the COVIDx-CXR-2 dataset to perform the image-only COVID-19 diagnosis task, and adopt the COVID-19-Delta data labelled in English to perform the text-only and image-text medical diagnosis tasks. We further select self-supervised learning and contrastive learning methods for comparison, i.e., CLIP^54^, ConVIRT^34^, and BioViL^30^. Since previous models had not attempted to deal with image-only, text-only and image-text tasks simultaneously, we re-implement these methods for evaluation.

Table 2 shows the diagnosis accuracy of our framework and the previous methods on COVID-19 classification, where our Med-MLLM achieves superior performance on all tasks and datasets. It not only achieves competitive results compared to previous methods with 1% training data, but also outperforms them when using 100% training data. The results demonstrate the validity of our method in relaxing the dependency on the high quality of labelled data for training, while making an accurate COVID-19 diagnosis.

**Table 2:** The diagnosis accuracy (AUC) of COVID-19 image-only, text-only and image-text disease classification experiments.

Methods	Year	Ratio of training data	Retrospective studies			Prospective studies		
			Image-only	Text-only	Image-text	Image-only	Text-only	Image-text
CLIP^54^	2021	1%	87.5	75.6	88.6	58.7	65.3	69.9
ConVIRT^34^	2022	1%	88.1	86.4	88.8	59.6	66.4	71.5
BioViL^30^	2022	1%	90.4	89.7	91.0	60.9	68.8	73.0
Med-MLLM	Ours	1%	95.3_(0.3)_	93.8_(0.5)_	95.9_(0.4)_	64.8_(1.1)_	72.9_(0.8)_	78.2_(0.7)_
CLIP^54^	2021	100%	95.7	83.3	89.0	63.5	68.8	75.2
ConVIRT^34^	2022	100%	97.6	94.5	97.7	70.4	77.6	82.1
BioViL^30^	2022	100%	97.4	94.5	98.2	66.7	80.5	84.4
Med-MLLM	Ours	100%	**98.4**_(0.2)_	**96.3**_(0.4)_	**98.7**_(0.2)_	**81.0**_(0.4)_	**84.1**_(0.5)_	**90.3**_(0.3)_

All values are reported in percentage (%). The best results are in bold.

#### Prospective studies

We pre-train the model on Delta data and fine-tune the model on Omicron data. As shown in Table 2, with 1% of Omicron data, our method can outperform several previous works (e.g., CLIP). More encouragingly, with 100% training labels, Med-MLLM surpasses the previous method by up to 10.6%, 3.6%, and 5.9% in diagnosis accuracy on image-only, text-only, and image-text classification tasks, respectively. The performance of prospective studies assesses the good generalisation capability of our approach in dealing with situations where the training data are scarce. Therefore our Med-MLLM is suitable for new pandemics caused by rapidly developing pathogens, improving the practical value of AI-based decision-support tools in clinical practice.

### COVID-19 prognosis

The COVID-19 prognosis task aims at predicting the survival of COVID-19 patients, i.e., predicting whether the patients will survive after treatment in the hospital. In this experiment, we evaluate the performance of prognosis on COVID-CXR and COVID-HCH datasets.

#### Retrospective studies

We conduct the image-only task on the COVID-CXR dataset and conduct the text-only and image-text tasks on the COVID-19-Delta data from the COVID-HCH dataset. Similar to the COVID-19 diagnosis task, we also re-implement the existing methods for COVID-19 prognosis. The results of COVID-19 prognosis are reported in Table 3, showing that our Med-MLLM is comparable to the previous approaches with 1% training data. Using the full training data, our method outperforms previous methods by up to 4.6%, 1.1%, and 1.5% in AUC on image-only, text-only, and image-text COVID-19 prognosis tasks, respectively.

**Table 3:** AUC values of COVID-19 prognosis experiments, which aim to predict the survival of COVID-19 patients.

Methods	Year	Ratio of training data	Retrospective studies			Prospective studies		
			Image-only	Text-only	Image-text	Image-only	Text-only	Image-text
CLIP^54^	2021	1%	70.4	84.3	89.5	66.9	76.7	81.3
ConVIRT^34^	2022	1%	75.3	88.1	92.6	70.6	81.2	85.4
BioViL^30^	2022	1%	77.1	89.0	92.9	70.8	82.1	85.7
Med-MLLM	Ours	1%	82.8_(0.5)_	92.1_(0.3)_	95.7_(0.3)_	81.2_(0.7)_	88.3_(0.8)_	92.0_(0.5)_
CLIP^54^	2021	100%	79.5	91.7	93.2	70.6	84.0	88.3
ConVIRT^34^	2022	100%	83.4	93.8	95.4	77.5	88.7	90.1
BioViL^30^	2022	100%	83.5	94.2	95.1	77.0	87.9	89.8
Med-MLLM	Ours	100%	**88.1**_(0.2)_	**95.3**_(0.2)_	**96.6**_(0.1)_	**85.7**_(0.4)_	**92.8**_(0.2)_	**94.9**_(0.2)_

All values are reported in percentage (%). The best results are in bold.

#### Prospective studies

We adopt the Omircon data to report the results of prospective studies. In implementations, we pre-train the model on Delta and predict for Omicron. The results illustrated in Table 3 indicate that when it comes to COVID-19 Omircon prognosis, with 1% of data for fine-tuning, our Med-MLLM surpasses existing methods by substantial margins demonstrating the effectiveness of our method in making an accurate and fast COVID-19 diagnosis with limited labelled data. With 100% training data, our method surpasses existing self-supervised learning and contrastive learning methods, which is in accordance with the results of COVID-19 reporting and diagnosis.

## Discussion

In addition to COVID-19, our Med-MLLM can be readily applied to other chest/respiratory diseases. Table 4 shows the performances of Med-MLLM on the CheXpert^36^, NIH ChestX-ray^69^, RSNA Pneumonia^70^, SIIM-ACR Pneumothorax^71^, and Shenzhen Tuberculosis^72^ benchmark datasets for common disease classification tasks. We follow previous works^30–32,34,73^ to pre-process the datasets and perform the evaluation. As we can see from Table 4, with limited labels (i.e., 1% of CheXpert, NIH ChestX-ray, RSNA Pneumonia, SIIM-ACR Pneumothorax datasets, and 10% of Shenzhen Tuberculosis), our method can achieve competitive results with previous fully-supervised methods trained on full labels. In particular, our Med-MLLM with 1% training data outperforms previous methods trained with 100% data on the CheXpert and RSNA datasets. Then, in Table 5, we further evaluate the performance of our method on 14 common thorax diseases. The t-tests between the results from Med-MLLM and the best-performing baseline REFERS indicate that the improvement is significant with *p* < 0.01. As we can see, our approach Med-MLLM (1%) achieves up to 0.4%, 0.5%, 0.1%, and 0.2% absolute improvements upon the current best results trained with full data for diseases–consolidation, effusion, infiltration, and pneumonia, respectively. More encouragingly, with all training labels as in previous works, our Med-MLLM (100%) can outperform these methods across all datasets and diseases. The promising results assess the generalisation capabilities of our approach.

**Table 4:** The diagnosis accuracy of different methods on various diseases across CheXpert, NIH ChestX-ray, RSNA Pneumonia, SIIM-ACR Pneumothorax, and Shenzhen Tuberculosis datasets.

Methods	Year	Ratio of training data	CheXpert	NIH ChestX-ray	RSNA	SIIM-ACR	Tuberculosis
ConVIRT^34^	2022	1% / 10%	87.0	66.2	88.8	71.3	93.7
BioViL^30^	2022	1% / 10%	86.8	69.5	88.1	69.5	95.0
REFERS^32^	2022	1% / 10%	87.2	76.7	89.4	76.6	95.8
Med-MLLM	Ours	1% / 10%	88.9_(0.5)_	83.3_(0.9)_	93.4_(0.5)_	87.5_(0.7)_	96.7_(0.4)_
ConVIRT^34^	2022	100%	88.1	81.3	92.7	90.0	96.4
BioViL^30^	2022	100%	87.9	82.5	89.1	86.9	97.1
REFERS^32^	2022	100%	88.2	84.7	92.7	89.3	98.0
Med-MLLM	Ours	100%	**89.5**_(0.2)_	**88.1**_(0.3)_	**95.3**_(0.2)_	**94.0**_(0.4)_	**98.6**_(0.1)_

All values are reported in percentage (%). The best results are in bold.

**Table 5:** The diagnosis accuracy on 14 common thorax diseases from the NIH ChestX-ray dataset.

Methods	Year	Ratio of training data	Atelectasis	Cardiomegaly	Consolidation	Edema	Effusion	Emphysema	Fibrosis	Hernia	Infiltration	Mass	Nodule	Pleural thickening	Pneumonia	Pneumothorax
NIH^69^	2017	1%	73.3	69.6	76.0	81.7	80.5	67.1	64.9	64.8	65.8	67.0	62.3	65.7	65.0	74.0
Context Restoration^105^	2019	1%	69.1	64.4	73.2	73.8	78.1	70.0	62.1	70.2	65.2	62.4	59.1	65.0	62.2	73.8
C2L^83^	2020	1%	75.1	67.1	77.6	75.1	83.4	71.5	66.8	70.0	63.8	70.1	66.2	68.1	65.7	74.4
Model Genesis^82^	2021	1%	72.1	67.1	75.8	76.1	80.6	72.6	64.8	73.5	65.7	65.2	62.2	67.6	64.8	76.2
TransVW^81^	2021	1%	74.5	68.9	76.7	79.8	81.1	67.9	68.7	68.2	66.8	66.5	66.2	68.5	68.8	75.0
REFERS^32^	2022	1%	77.5	85.6	78.6	84.9	85.4	79.5	72.3	77.1	67.5	76.2	66.5	71.6	69.3	81.7
Med-MLLM	Ours	1%	80.1	88.2	82.5	89.4	89.2	90.4	80.0	87.8	74.2	81.6	75.9	77.9	77.2	87.3
NIH^69^	2017	100%	78.3	89.3	77.6	87.9	85.9	87.4	78.5	88.8	65.9	79.9	70.7	74.5	71.0	84.7
Context Restoration^105^	2019	100%	75.8	82.9	76.4	86.6	84.8	88.2	78.6	83.0	70.0	79.6	69.5	73.2	69.4	84.0
C2L^83^	2020	100%	81.1	90.2	81.0	88.1	88.0	88.3	80.8	86.8	72.0	82.7	74.1	76.2	75.3	85.9
Model Genesis^82^	2021	100%	78.8	84.5	79.2	87.8	86.6	89.7	81.0	85.2	71.1	81.9	73.2	75.8	73.0	85.6
TransVW^81^	2021	100%	79.8	85.0	80.0	88.2	87.1	90.1	81.8	85.9	72.3	82.6	74.4	76.6	74.0	86.1
REFERS^32^	2022	100%	83.0	92.3	82.1	90.2	88.7	91.4	83.9	93.3	74.1	85.5	76.7	78.5	77.0	89.1
Med-MLLM	Ours	100%	**85.4**	**94.1**	**84.7**	**91.3**	**90.2**	**95.0**	**88.2**	**94.6**	**76.9**	**88.7**	**79.3**	**82.8**	**79.1**	**89.9**

All values are reported in percentage (%). The best results are in bold.

To further evaluate the effectiveness of our framework for rare diseases, we assess the diagnosis performances of existing LLMs, i.e., GPT-2, GPT-3, ChatGPT (GPT-3.5 version), and GPT-4^28,74^ that are released by OpenAI. Since LLMs only accept the text as input, we perform the text-only COVID-19 diagnosis task, which aims to distinguish COVID-19 from non-COVID-19 cases. To obtain the diagnosis accuracy (i.e., disease classification performance) from the LLMs, we take the following text as input: ‘Original Clinical Text’ + ‘Is this a COVID-19 case?’. Then, we sample the probabilities of ‘Yes’ (*P*_yes_) and ‘No’ (*P*_no_) from the next predicted token by GPT. Finally, if *P*_yes_ > *P*_no_, we take the ‘Yes’ as the output of LLMs; if *P*_yes_ < *P*_no_, we take the ‘No’ as the output of LLMs. In this way, we can obtain the COVID-19 diagnosis accuracy of LLMs. For the ChatGPT and GPT-4, we follow previous works^75,76^ to incorporate the few-shot prompting^28^ and chain-of-thought prompting^77^ strategies. It means that we incorporate five examples, which cover both COVID-19 and non-COVID-19 cases, and instructions as input to request them to generate the response. Therefore, the full input is:

*This is just a text classification test. Analyze the report first, then provide the final answer here based on the following examples, which must be either “Yes" or “No"*.

*Report: ‘Original Clinical Text’;*

*Question: Is this a COVID-19 case?*

*Answer: Provide the final answer here, which must be either “Yes" or “No"*.

At last, due to the potential variation in output from ChatGPT, we conduct five runs for each enquiry and select the answer that appears most frequently as the final answer. In addition, it can also be considered as an ensemble approach to achieve better results. Table 6 reports the performances of our method and existing strong LLMs. As we can observe, our approach performs better than several strong LLMs, i.e., GPT-2, GPT-3, and ChatGPT, and achieves a competitive result w.r.t. GPT-4. It is worth noting that although these LLMs have shown great success in natural text understanding, we cannot directly adopt the results provided by ChatGPT in the medical domain^78,79^.

**Table 6:** Comparison with existing large language models (LLMs), i.e., GPT-2, GPT-3, ChatGPT (GPT-3.5), and GPT-4.

Methods	GPT-2	GPT-3	ChatGPT	GPT-4	Med-MLLM
COVID-19 diagnosis	87	91	93	98	97

We perform the text-only COVID-19 diagnosis task to compare the usefulness of our approach with that of the strong LLMs in the medical domain. All values are reported in percentage (%).

We perform a robustness analysis to examine whether our method can aid in the COVID-19 diagnosis of new regions by predicting the COVID-19 cases in new regions. To this end, we conduct a cross-region prediction by training the methods on patient data from one region and evaluating the methods on patient data from other regions. In implementations, the BIMCV-COVID-19 dataset collected in Spain, the COVID-HCH dataset collected in China, and the COVID-CXR dataset collected in over 20 countries (excluding Spain and China) are used for the validation. The image-only COVID-19 diagnosis accuracy of our method and previous methods are summarised in Table 7. It shows that our approach consistently outperforms previous methods and achieves solid performances in COVID-19 diagnosis in new regions. In particular, when transferring our approach trained on patient data from Spain to China, we observe an encouraging performance, i.e., 90.1% AUC, which is competitive with the region-specific results of previous works CLIP (80.7% AUC) and BioViL (90.4% AUC), which were obtained by training and testing on the data collected from the same region. Similarly, the cross-region performance of the Spain region (84.8% AUC) of our method, which is trained on China, is competitive with the region-specific result of CLIP (85.4% AUC). These results highlight the transferability and robustness of our approach, leading to a higher-quality diagnosis of rare diseases in new regions than the current methods.

**Table 7:** Robustness analysis aims to examine whether our framework can provide COVID-19 decision support for new regions.

Training regions	Methods	Year	Testing Regions	
			Spain	China
Spain (BIMCV-COVID-19)	CLIP^54^	2021	85.4	75.4
	ConVIRT^34^	2022	92.7	85.6
	BioViL^30^	2022	92.8	83.3
	Med-MLLM	Ours	**95.2**	**90.1**
China (COVID-HCH)	CLIP^54^	2021	70.5	80.7
	ConVIRT^34^	2022	79.9	91.7
	BioViL^30^	2022	77.0	90.4
	Med-MLLM	Ours	**84.8**	**93.9**
>20 Countries (COVID-CXR	CLIP^54^	2021	63.3	61.5
excl. Spain & China)	ConVIRT^34^	2022	71.2	69.0
	BioViL^30^	2022	69.6	70.4
	Med-MLLM	Ours	**78.2**	**74.8**

We perform cross-region prediction by training on patient data from one region and evaluating on patient data with different phenotypes from other regions. All values are reported in percentage (%). The best results are in bold.

**Fig. 4:**
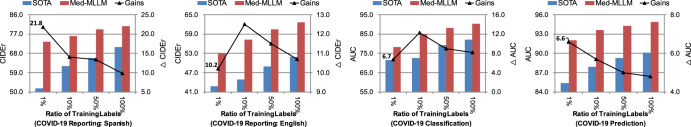
Results of Med-MLLM and state-of-the-art (SOTA) methods with respect to the increasing quantity of training labels. The margins in different ratios are shown with the polyline. As we can see, our method can be continuously improved using more training labels which may be available as the disease evolves.

To further assess the effectiveness of our approach in diagnosis, we present to use more labels to conduct continuous learning to train the model continuously. It can evaluate whether the model can continue to be improved when more labelled data are collected as the disease evolves. It is particularly useful in real-world settings. To this end, in Fig. 4, we evaluate the performance of Med-MLLM with respect to the increasing quantity of training labels. Specifically, we evaluate the results on the BIMCV-COVID-19 and COVID-Omicron data for COVID-19 reporting, diagnosis, and prognosis tasks across modalities, languages, and regions. For comparison, we also re-implement the state-of-the-art (SOTA) models (i.e., XProNet^63^ for reporting and ConVIRT^34^ for diagnosis and prognosis) using the same training labels to better understand the strengths of our method. We conduct multiple runs with different seeds and report the average performance. As we can see in Fig. 4, for different COVID-19 decision-support tasks, our method Med-MLLM consistently outperforms SOTA with the different numbers of training labels. With more training labels, our method can be continuously improved. It is worth noting that, under the low label setting, e.g., 1% of training labels, our approach surpasses the SOTA by large margins, up to 21.8%, 6.7%, and 6.6% absolute improvements on COVID-19 reporting, diagnosis, and prognosis tasks, respectively. More importantly, with 10% labelled data for training, our method can outperform previous SOTA methods trained with 100% training data. It demonstrates the effectiveness of our approach in relaxing the reliance on the annotations to provide a solid basis for COVID-19 decision-support, which is particularly useful for rare diseases, where the labels are scarce at the early stage.

**Fig. 5:**
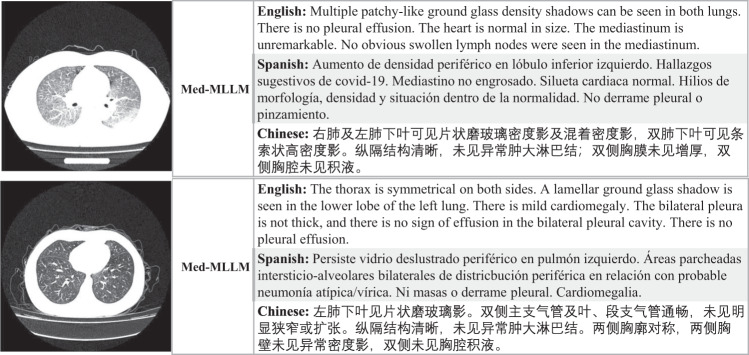
The examples of COVID-19 reports generated by our Med-MLLM framework for different languages, i.e., English, Spanish, and Chinese. As we can see, Med-MLLM can generate accurate and informative reports across different languages to relieve the heavy burden of physicians and could support them in clinical decision-making.

We provide two intuitive examples to illustrate our approach. Figure 5 shows that our method Med-MLLM can simultaneously generate useful and informative reports across different languages. More importantly, Med-MLLM is able to accurately report important abnormalities, e.g., ‘*multiple patchy-like ground glass density shadow*’ in the first example, and ‘*a lamellar ground glass shadow is seen in the lower lobe of the left lung*’ in the second example. It is encouraging that our approach can accurately report abnormalities. Overall, with limited labels, our Med-MLLM can generate informative and “believable” reports for different languages, demonstrating its capability for combating rare diseases.

We further detect the hallucinations and missing facts in the generated reports. To successfully assist physicians and reduce their workloads of writing medical reports, it is important to generate accurate reports (*faithfulness* or precision), such that the model does not generate hallucinations that “do not exist”. It is also necessary to provide comprehensive facts (*comprehensiveness* or recall), i.e., the model does not leave out the true findings. To this end, we first employ a medical natural language processing (NLP) tool from the work of CheXpert^36^, to label the ground truth reports, e.g., [Abnormality_A, Abnormality_B]. Then, we again employ the NLP tool to label the generated reports, e.g., [Abnormality_B, Abnormality_C]. We can find that the model generates a hallucination, i.e., [Abnormality_C], and misses a fact, i.e., [Abnormality_A]. Therefore, we can use this method to calculate the ‘Precision’ and ‘Recall’ scores to preliminary detect the hallucinations and missing facts, respectively. At last, we further calculate the F1 score to obtain the overall performance. Since the NLP tool can extract abnormalities from the English text, we conduct the evaluation on English report generation. For comparison, we also calculate the Precision, Recall, and F1 scores of previous methods, i.e., R2Gen^61^, KGAE^62^, and XProNet^63^. For a fair comparison, both previous methods and our method are trained on 100% of training data. The results are reported in Table 8, showing that our Med-MLLM method surpasses previous methods on all metrics by 5.5%, 3.6%, and 4.6% in terms of Precision, Recall, and F1 scores, respectively. It shows that our approach can generate more faithful reports (i.e., fewer hallucinations) and more comprehensive reports (i.e., fewer missing facts) than previous methods, demonstrating that our method can better assist physicians in reducing their workload.

**Table 8:** We detect the hallucinations and missing facts in reports generated by different methods.

Methods	Year	Precision	Recall	F1
R2Gen^61^	2020	71.8	82.0	76.6
KGAE^62^	2021	70.5	79.8	74.9
XProNet^63^	2022	73.6	84.7	78.8
Med-MLLM	Ours	**79.1**	**88.3**	**83.4**

Higher precision and recall indicate fewer hallucinations and missing facts, respectively. Therefore, higher is better for all metrics. All values are reported in percentage (%). The best results are in bold.

To better understand the effectiveness of each introduced component, we provide a thorough ablation study of our Med-MLLM in Table 9. It shows that all of our introduced components can bring improvements to downstream tasks. In detail, as image-only pre-training can enable the model to learn broad thorax knowledge, e.g., the diagnostic information, from visual images, removing it would impair the performances (i.e., 73.4% → 57.8% in CIDEr on reporting, 78.2% → 69.4% in AUC on diagnosis, 92.0% → 82.3% AUC on prognosis). The impaired performances assess the effectiveness of learning the important visual characteristics from medical images to support accurate diagnosis and prognosis. Besides, we find that removing the patient-level contrastive learning (PCL) impairs performance across all tasks. By comparing settings (c-e), we notice that, among the introduced three modules in text-only pre-training, the sentence reconstruction module (SR), which can help the model efficiently learn to generate reports, brings the most improvements on reporting. In contrast, the other two modules, MLM and FIA, result in more improvements on diagnosis and prognosis. The image-text pre-training aims to unify the learned medical knowledge from medical images and text. The performance across all tasks decreases when it is removed, showing that unifying visual and textual information can boost the representation of medical data. Overall, the ablation study demonstrates the effectiveness of the Med-MLLM, where all the components can contribute to performance.

**Table 9:** Ablation study of the proposed components in three pre-training settings: image-only, text-only, and image-text.

Settings	Methods	Reporting: Spanish					Reporting: English					Diagnosis	Prognosis
		BLEU-2	BLEU-3	BLEU-4	ROUGE-L	CIDEr	BLEU-2	BLEU-3	BLEU-4	ROUGE-L	CIDEr	Image-Text	Image-Text
Full	Med-MLLM	47.6	42.0	38.1	55.4	73.4	32.4	28.7	24.9	46.7	53.0	78.2	92.0
(a)	w/o image-only (PCL)	45.8	39.9	34.7	54.3	70.9	31.2	27.5	23.0	45.3	51.8	75.7	90.2
(b)	w/o image-only	39.5	35.4	32.2	48.7	57.8	28.1	24.0	19.3	42.1	45.9	69.4	82.3
(c)	w/o text-only (MLM)	45.7	39.6	34.2	54.7	71.1	31.3	27.2	21.8	44.9	50.7	73.3	86.7
(d)	w/o text-only (SR)	44.2	38.5	33.6	54.1	70.3	30.2	26.7	21.5	44.0	49.4	76.3	89.1
(e)	w/o text-only (FIA)	46.0	40.1	36.5	54.2	70.6	31.8	27.5	23.2	45.5	51.6	75.4	88.3
(f)	w/o image-text	42.4	38.3	34.2	53.7	66.5	29.8	25.6	20.7	44.5	48.9	74.1	85.4

We perform the analysis on COVID-19 reporting, diagnosis, and prognosis. All values are reported in percentage (%).

At last, to explore the effect of scaling up the number of model parameters, we introduce a larger version of the language model (i.e., Med-MLLM-Large) with 8.9 billion parameters initialized with GatorTron^80^, where the number of layers is 56, the number of attention heads is 56, and the dimensionality is 3584. For comparison, we perform the evaluation on the text-only COVID-19 diagnosis and prognosis tasks to evaluate the performance of different language models. The results in Table 10 show that the Med-MLLM-Large has better performance than the Med-MLLM-Base by 1.7 ~ 3.6 in AUC values. It not only shows that more model parameters can lead to further improvements, but also demonstrates the potential of LLM that can be further improved in the future by directly scaling up the models.

**Table 10:** The COVID-19 diagnosis and prognosis accuracy (AUC) of different sizes of our Med-MLLM, which are trained on full training data.

Methods	Year	Retrospective studies		Prospective studies	
		Diagnosis	Prognosis	Diagnosis	Prognosis
ClinicalBERT^94^	2019	93.2	93.4	74.8	87.5
BioBERT^93^	2020	85.9	92.2	71.2	84.6
PubMedBERT^92^	2022	95.7	93.9	76.7	88.4
Med-MLLM-Base	Ours	96.3	95.3	84.1	92.8
Med-MLLM-Large	Ours	**98.0**_(+1.7)_	**97.2**_(+1.9)_	**87.7**_(+3.6)_	**94.9**_(+2.1)_

All values are reported in percentage (%). The best results are in bold.

## Methods

In this section, we describe in detail the three main components of our deep learning model.

### Image-only pre-training

We first introduce Patient-level Contrastive Learning (Fig. 2a) and then present the image augmentation and regularisation.

#### Patient-level contrastive learning

We conduct image-only pre-training to learn medical knowledge from the large-scale unlabelled image-only data. Several existing works based on self-supervised learning or contrastive learning^81–83^ have shown the effectiveness of training models on large-scale image-only medical data. In this work, inspired by the success of contrastive learning in natural images^45,46,56^, we introduce Image-level Contrastive Learning (ICL) and Patient-level Contrastive Learning (PCL) for medical image understanding.

In implementations, for a fair comparison, we choose ResNet-50^55^ as our basic model to perform the image-only training, while several works^84^ are based on more powerful models, i.e., Vision Transformer (ViT)^85^. During training, we first sample a mini-batch of *N* medical images. Then, for each input medical image, we randomly select the image augmentation functions, e.g., affine transformations (shearing and rotation), colour jittering (contrast and brightness), and random Gaussian blurring^30,34,45,46,56^, to transform the current medical image into two correlated views of the same image, encoded by ResNet-50 as *V*_*i*_ and *V*_*j*_, which we consider as a positive pair. As a result, we can obtain a training batch with 2*N* images. We treat the other 2(*N* − 1) augmented images as negative examples to *V*_*i*_. The image-level contrastive learning aims to minimise the distance between positive examples, e.g., *V*_*i*_ and *V*_*j*_, while maximising the distance between negative examples, e.g., *V*_*i*_ and *V*_*k*_ (*k* ≠ *i*, *j*). To this end, we adopt the ICL loss to train our approach, defined as follows:

1 $${\ell }^{{{{\rm{ICL}}}}}=\mathop{\sum}\limits_{(i,j)}-\log \frac{\exp \left(\langle {V}_{i},{V}_{j}\rangle /\tau \right)}{{\sum }_{k\ne i}\exp \left(\langle {V}_{i},{V}_{k}\rangle /\tau \right)},$$

where the 〈 ⋅ , ⋅ 〉 denotes the cosine similarity and *τ* is a temperature hyperparameter^46^.

While conventional (image-level) contrastive learning can enable the model to understand the input medical images by training the model to distinguish whether the inputted medical images are from the same image or not, it is plausible that this could result in a model that is primarily learning to distinguish images based on the appearance of images, instead of the pathology. To incorporate the characteristics of medical images, we further introduce patient-level contrastive learning (PCL)^86,87^. In detail, PCL takes two images with completely different views, i.e., Anteroposterior (AP) and Posteroanterior (PA), as input. This approach prevents the model from distinguishing the input images by learning to capture the appearance. Instead, it forces the model to pay more attention to capturing the pathologies in medical images.

In implementations, PCL considers two medical images, e.g., AP and PA views, which are encoded by ResNet-50 as ${V}_{i}^{{\prime} }$ and ${V}_{j}^{{\prime} }$ from the same patient as a positive pair, and the remaining images from other patients in the mini-batch as negative examples. The PCL is defined as follows:

2 $${\ell }^{{{{\rm{PCL}}}}}=\mathop{\sum}\limits_{(i,j)}-\log \frac{\exp (\langle {V}_{i}^{{\prime} },{V}_{j}^{{\prime} }\rangle /\tau )}{{\sum }_{k\ne i}\exp (\langle {V}_{i}^{{\prime} },{V}_{k}^{{\prime} }\rangle /\tau )}.$$

The full training objective of image-only pre-training is defined as: *ℓ*_Image_ = *ℓ*^PCL^ + *ℓ*^ICL^. As we can see, the training of our method does not rely on labelled data, thus, the image-only pre-training could be unsupervised. During training, we exploit the image-only data from several public datasets, including CheXpert^36^, MIMIC-CXR^37^, COVID-CXR^39,41^, COVID-19-CT-CXR^43^, COVIDx-CXR-2^40^, BIMCV-COVID-19^42^, RSNA Pneumonia^70^, and COVID-19 CT^44^, to conduct the image-only pre-training. As a result, we can learn comprehensive thorax knowledge from image-only data. In particular, when we evaluate the Med-MLLM on a dataset, we will exclude it from the pre-training set.

#### Image augmentation and regularisation

Since the size of medical image datasets is usually smaller than the size of natural image datasets, such as ImageNet^88^, we adopt image augmentation strategies to further improve the performance and robustness of our framework. In implementations, we apply random cropping, rotation (−10 to 10 degrees), brightness and contrast adjustment with ratios randomly sampled from [0.8, 1.2], horizontal flipping with 50% probability, and Gaussian blurring with *σ* ∈ [0.1, 3.0], as used in previous works^30,32,34,89^. Besides augmentation, we introduce several regularisation methods into our framework. First, we re-write the full training loss of the image-only pre-training as follows:

3 $${\ell }_{{{{\rm{Image}}}}}=\lambda {\ell }^{{{{\rm{PCL}}}}}+(1-\lambda ){\ell }^{{{{\rm{ICL}}}}},$$

where *λ* ∈ [0, 1] is the hyperparameter that controls the regularisation. We set *λ* = 0.2 according to the performance on the validation set. Meanwhile, the global batch normalisation^46^, layer normalisation^90^ and dropout^91^ are used in regularisation. The experiments show that all the introduced modules contribute to improved performances.

### Text-only pre-training

As shown in Fig. 1, we adopt a specialised medical large language model (LLM) with a radiology-specific vocabulary. Meanwhile, as shown in Fig. 2b, we present two training objectives and a text augmentation method to enhance the performance of our LLM.

#### Large language model (LLM)

In recent years, several efforts^75,76,80,92^ have been invested to build medical large language models, which have shown great success in processing medical text, such as BioBERT^93^, ClinicalBERT^94^, BlueBERT^95^, and PubMedBERT^92^. In detail, BioBERT is pre-trained on PubMed^47^, ClinicalBERT is pre-trained on MIMIC-III^48^, while BlueBERT combines both corpora for pre-training. All these methods use a vocabulary defined on open-domain text (i.e., Wiki + Books) as in original BERT^49^. For comparison, PubMedBERT is pre-trained on PubMed^47^ with a medical vocabulary designed on medical text from PubMed.

As we can see, among the above models, only PubMedBERT designed a domain-specific vocabulary for training; none of the existing LLMs designed a radiology-specific vocabulary. For example, the radiology-specific term ‘cardiomegaly’ will be broken into multiple sub-words (word pieces), i.e., ‘card-io-me-gal-y’ and ‘cardio-me-gal-y’ in the ClinicalBERT and PubMedBERT, respectively. Since most sub-words have no medical relevance, it hinders the LLMs from accurately understanding the radiology-specific medical terms^30^.

To resolve this, we introduce a radiology-specific vocabulary^30^ based on the medical texts from PubMed^47^, MIMIC-III clinical notes^48^, and MIMIC-CXR medical reports^37^. Based on the designed radiology-specific vocabulary that includes the whole-word radiology-specific terms (e.g., ‘cardiomegaly’), we perform pre-training of our model on the text-only data from PubMed + MIMIC-III + MIMIC-CXR corpora. In the following, we will introduce the training objectives of our framework in detail.

#### Training objectives

This section introduces the training objectives used in our method. In implementations, we adopt three training objectives, i.e., Masked Language Modelling (MLM), Sentence Reconstruction (SR), and medical-report-specific Findings-Impression Alignment (FIA).

*Masked Language Modelling (MLM)*. Given a mini-batch of *N* medical text sequences, following conventional BERT^49,50^, for each medical text sequence, we randomly mask out the input words with 15% probability, resulting in *N* sequences of masked words and unmasked words (*w*_m_, *w*_\m_). The training objective of MLM is to predict the randomly masked words *w*_m_ based on the remaining unmasked words *w*_\m_. Therefore, the MLM loss is defined as:

4 $$\begin{array}{r}{\ell }^{{{{\rm{MLM}}}}}=-\frac{1}{N}\mathop{\sum}\limits_{({w}_{{{{\rm{m}}}}},{w}_{\backslash {{{\rm{m}}}}})}\log \left(p\left({w}_{{{{\rm{m}}}}}| {w}_{\backslash {{{\rm{m}}}}}\right)\right),\end{array}$$

where *p* denotes the predicted probability. The masked tokens are predicted as a classification problem by selecting one token from the vocabulary.

*Sentence Reconstruction (SR)*. We further introduce a training objective, sentence reconstruction, to boost the understanding and generation of medical text. As shown in Fig. 2b, we introduce an additional text decoder to reconstruct the input medical text in the auto-encoding pipeline. It means that the decoder takes the input medical text as the ground truth, i.e., *W* = {*w*_1_, *w*_2_, …, *w*_*M*_}, for sentence reconstruction. Therefore, the sentence reconstruction loss is defined as:

5 $${\ell }^{{{{\rm{SR}}}}}=-\frac{1}{N}\mathop{\sum}\limits_{W}\mathop{\sum }\limits_{t=1}^{M}\log \left(p\left({w}_{t}| {w}_{1:t-1}\right)\right).$$

The training objective is to reconstruct the same input sentence, and it is straightforward for our model to be trained^51,62,96^ to learn the necessary domain knowledge from the unlabelled medical texts.

*Findings-Impression Alignment (FIA)*. We observe that a medical report contains rich structural information. Typically, it contains a section for “findings” and another section for “impression”, where the former is a paragraph of multiple sentences describing both the normal and abnormal findings in detail, and the latter summarizes a diagnostic conclusion from the findings section. We therefore introduce the training objective FIA^30^ to exploit the structural information of medical reports.

In implementations, we adopt self-supervised learning and contrastive loss^46^. We first sample a batch of *N* medical reports, including *N* pairs of “Findings” and “Impression” sections. Then, we denote the encoded “Findings” and “Impression” sections of the *i*th input medical report as (${T}_{i}^{F},{T}_{i}^{I}$), which we consider as a positive pair. “Findings” and “Impression” from different medical reports are used as negative pairs. The training loss of FIA is defined as follows:

6 $$\begin{array}{r}{\ell }_{i}^{(F\to I)}=-\log \frac{\exp \left(\left\langle {T}_{i}^{F},{T}_{i}^{I}\right\rangle /\tau \right)}{\mathop{\sum }\nolimits_{j = 1}^{N}\exp \left(\left\langle {T}_{i}^{F},{T}_{j}^{I}\right\rangle /\tau \right)},\\ {\ell }_{i}^{(I\to F)}=-\log \frac{\exp \left(\left\langle {T}_{i}^{I},{T}_{i}^{F}\right\rangle /\tau \right)}{\mathop{\sum }\nolimits_{j = 1}^{N}\exp \left(\left\langle {T}_{i}^{I},{T}_{j}^{F}\right\rangle /\tau \right)},\end{array}$$

where the 〈 ⋅ , ⋅ 〉 denotes the cosine similarity and *τ* is a temperature hyperparameter^46^. We note that the numerators in both two losses are equal, representing the similarity between ${T}_{i}^{F}$ and ${T}_{i}^{I}$ for the *i*th positive pair of “Findings” and “Impression”. However, their denominators differ. For the first loss ${\ell }_{i}^{(F\to I)}$, the denominator measures the similarity between the *i*th “Findings” $\left({T}_{i}^{F}\right)$ and all other “Impressions”. For ${\ell }_{i}^{(I\to F)}$, the denominator measures the similarity between the *i*th “Impression” $\left({T}_{i}^{I}\right)$ and all other “Findings”. Therefore, the two Equations are distinct and respectively reflect the similarity of “Findings” relative to “Impression” (*F* → *I*) and “Impression” relative to “Findings” (*I* → *F*).

Finally, we obtain the full training objective of FIA by combining the ${\ell }_{i}^{(F\to I)}$ and ${\ell }_{i}^{(I\to F)}$, as follows:

7 $${\ell }^{{{{\rm{FIA}}}}}=\frac{1}{N}\mathop{\sum }\limits_{i=1}^{N}\left({\ell }_{i}^{(F\to I)}+{\ell }_{i}^{(I\to F)}\right).$$

Through the above operation, our method exploits the structural information to improve the understanding of medical texts, and thus boost the performance.

#### Text augmentation and regularisation

To further improve the performance of our method, we present a text augmentation method and several regularisation methods.

For the text augmentation, we observe that each medical text is composed of multiple sentences, which are usually permutation-invariant^97^. Therefore, we can randomly shuffle the sentences to augment the medical texts to boost performance.

Meanwhile, we introduce *α* and *β* for better regularisation. The full training objective of text-only pre-training *ℓ*_Text_ is defined as follows:

8 $${\ell }_{{{{\rm{Text}}}}}={\ell }^{{{{\rm{FIA}}}}}+\alpha {\ell }^{{{{\rm{SR}}}}}+\beta {\ell }^{{{{\rm{MLM}}}}}.$$

In implementations, the *α* and *β* are set to 0.5 and 0.1, respectively, according to the performances on the validation set. In detail, our framework is first trained using MLM (*ℓ*^MLM^), then is trained using the combination of MLM and FIA, and finally is trained on the full training objective *ℓ*_Text_.

### Image-text pre-training

Most recently, several image-text pre-training methods^30,32,34,44^ have been proposed to demonstrate the importance of unifying the images and texts to improve the understanding of medical data. However, all existing methods mainly adopt supervised training and heavily rely on large-scale coupled image-report pairs for training, while collecting labelled and paired medical data across different modalities is typically very costly and time-consuming. To this end, we introduce the image-text pre-training to relax the reliance on the labelled image-text pairs^89^.

#### Soft image-text alignment (SITA)

As shown in Fig. 2 (c), we incorporate a knowledge base and a pre-training objective, i.e., Soft Image-Text Alignment (SITA)^89,98^. In particular, given a mini-batch of *N* randomly sampled pairs of images and texts, we adopt MetaMap^99^ to extract entities defined in the Unified Medical Language System (UMLS)^53^ from the *i*th medical text. Following previous works^36,37,62,89,100,101^, we focus on the 14 common radiographic entities (Atelectasis, Cardiomegaly, Consolidation, Edema, Enlarged Cardiomediastinum, Fracture, Lung Lesion, Lung Opacity, No Finding, Pleural Effusion, Pleural Other, Pneumonia, Pneumothorax, Support Devices). As a result, given the medical text, e.g., *"A right pleural effusion. Heart size is enlarged. No evidence of pneumothorax”*, we can extract two entities, *pleural effusion* and *cardiomegaly*. Then, we construct a multi-hot vector ${H}_{i}^{T}$ of dimension 14 from the extracted entities, where 1/0 denotes the presence/absence of the radiographic entity. Similarly, for the *j*th medical image with diagnosis labels, we again adopt MetaMap^99^ to extract radiographic entities by mapping the raw diagnosis labels of medical images to UMLS concepts, e.g., “Normal” will be mapped to “No Findings”. As a result, the images and the texts can share the same radiographic entities. Then, we can construct a multi-hot vector ${H}_{j}^{V}$ of dimension 14 for the image. At last, we calculate the cosine similarity of ${H}_{i}^{T}$ and ${H}_{j}^{V}$ to measures the similarity of the *i*th text and the *j*th image. In this way, we measure the similarity between any text and image. The target similarity score ${s}_{ij}^{(T\to V)}$ between the *i*th text and the *j*th image is calculated as:

9 $${s}_{ij}^{(T\to V)}=\frac{\exp \left(\left\langle {H}_{i}^{T},{H}_{j}^{V}\right\rangle /\tau \right)}{\mathop{\sum }\nolimits_{k = 1}^{N}\exp \left(\left\langle {H}_{i}^{T},{H}_{k}^{V}\right\rangle /\tau \right)},$$

where 〈 ⋅ , ⋅ 〉 denotes the cosine similarity and *τ* is a temperature parameter. Similarly, we can obtain the target similarity score ${s}_{ji}^{(V\to T)}$ between the *j*th image and the *i*th text:

10 $${s}_{ji}^{(V\to T)}=\frac{\exp \left(\left\langle {H}_{j}^{V},{H}_{i}^{T}\right\rangle /\tau \right)}{\mathop{\sum }\limits_{k=1}^{N}\exp \left(\left\langle {H}_{j}^{V},{H}_{k}^{T}\right\rangle /\tau \right)}.$$

${s}_{ij}^{(T\to V)}$ and ${s}_{ji}^{(V\to T)}$ are used as the soft target labels of image-text alignment in the image-text pre-training, which will be introduced as follows.

To perform the image-text pre-training, we first use the BERT^49^ and ResNet-50^55^ to encode the *i*th text and *j*th image, resulting in *T*_*i*_ and *V*_*j*_, respectively. Therefore, the predicted similarity score ${{s}_{ij}^{{\prime} }}^{(T\to V)}$ between the *i*th text and the *j*th image and the predicted similarity score ${{s}_{ji}^{{\prime} }}^{(V\to T)}$ between the *j*th image and the *i*th text are calculated by:

11 $$\begin{array}{rcl}{{s}_{ij}^{{\prime} }}^{(T\to V)}&=&\frac{\exp (\langle {T}_{i},{V}_{j}\rangle /\tau )}{\mathop{\sum }\limits_{k=1}^{N}\exp (\langle {T}_{i},{V}_{k}\rangle /\tau )},\\ {{s}_{ji}^{{\prime} }}^{(V\to T)}&=&\frac{\exp (\langle {V}_{j},{T}_{i}\rangle /\tau )}{\mathop{\sum }\limits_{k=1}^{N}\exp (\langle {V}_{j},{T}_{k}\rangle /\tau )}.\end{array}$$

At last, the soft image-text alignment (SITA) loss is implemented by the cross entropy loss:

12 $$\begin{array}{rcl}{\ell }_{i}^{T\to V}&=&-\mathop{\sum }\limits_{j=1}^{N}{s}_{ij}^{(T\to V)}\log {{s}_{ij}^{{\prime} }}^{(T\to V)},\\ {\ell }_{j}^{V\to T}&=&-\mathop{\sum }\limits_{i=1}^{N}{s}_{ji}^{(V\to T)}\log {{s}_{ji}^{{\prime} }}^{(V\to T)},\\ {\ell }^{{{{\rm{SITA}}}}}&=&\frac{1}{N}\mathop{\sum }\limits_{k=1}^{N}\left({\ell }_{k}^{T\to V}+{\ell }_{k}^{V\to T}\right).\end{array}$$

Through the SITA, our method performs image-text pre-training to exploit unpaired medical images and texts to efficiently and accurately align medical data across modalities^89^.

#### Data augmentation and regularisation

Similarly, we introduce image augmentation in image-only pre-training and text augmentation in text-only pre-training to further boost the robustness and thus improve the performance of our method.

More importantly, during the regularisation, we incorporate the MLM loss for joint training, resulting in the full training objective of image-text pre-training as follows:

13 $${\ell }_{{{{\rm{Image}}}}-{{{\rm{Text}}}}}={\ell }^{{{{\rm{SITA}}}}}+\gamma {\ell }^{{{{\rm{MLM}}}}}.$$

In implementations, *γ* controls the regularisation and is set to 2, according to the performances on the validation set. Our preliminnarly experiments show the effectiveness of performing continuous MLM optimisation.

### Experiment settings

For a fair comparison, we adopt the ResNet-50^55^ as the image encoder and the BERT^5,102^ as the text encoder. The number of encoder layers is set to 6 and the dimension of the latent states is 768 unless otherwise stated. Meanwhile, we also explored a larger version of the language model^49,80^ with 8.9 billion parameters, where the number of layers is 56, the number of attention heads is 56, and the dimensionality of the latent states is 3584. We adopt the AdamW optimiser^103^ for training. We train our model in the order of image-only, text-only, and image-text pre-training. During image-only/text-only/image-text pre-training: the hyper-parameter *τ* is set to 0.5/0.5/0.1 according to the average performances on the validation sets; we use a learning rate of 10^−3^/2 × 10^−5^/5 × 10^−5^ and a batch size of 256/256/100. During fine-tuning, we use a batch size of 32/64/16 and a learning rate of 10^−4^ for parameter optimisation on the COVID-19 reporting/diagnosis/prognosis task. Our code is implemented in PyTorch^104^. During testing, we add a text decoder, i.e., Transformer^5^, to perform the reporting task, and add a fully connected layer to perform the diagnosis and prognosis tasks.

#### Ethical considerations

Our study was conducted on thirteen datasets, in which all Protected Health Information (PHI), e.g., patient name, sex, gender, and date of birth, is officially de-identified for all datasets used in our experiments. It means that the deletion of PHI from structured data sources (e.g., database fields that provide age, genotypic information, past and current diagnosis and treatment categories) is performed in compliance with the Health Insurance Portability and Accountability Act (HIPAA) standards in order to facilitate public access to the datasets.

#### Recruitment statement

We do not recruit any new human research participants for this study. For the public data, all necessary patient/participant consent has been obtained and the appropriate institutional forms have been officially archived.

### Reporting summary

Further information on research design is available in the Nature Research Reporting Summary linked to this article.

### Supplementary information

Reporting Summary

## Data Availability

The data used in our work may be available for research purposes from the corresponding authors upon reasonable request. 1) CheXpert is available at https://stanfordmlgroup.github.io/competitions/chexpert/. 2) COVIDx-CXR-2 is available at https://alexswong.github.io/COVID-Net/. 3) MIMIC-CXR is available at https://physionet.org/content/mimic-cxr/2.0.0/. 4) COVID-19-CT-CXR is available at https://github.com/ncbi-nlp/COVID-19-CT-CXR. 5) COVID-19 CT is available at https://covid19ct.github.io/. 6) COVID-CXR is available at https://github.com/ieee8023/covid-chestxray-dataset. 7) BIMCV-COVID-19 is available at https://bimcv.cipf.es/bimcv-projects/bimcv-covid19/. 8) PubMed is available at https://pubmed.ncbi.nlm.nih.gov/download/. 9) MIMIC-III is available at https://physionet.org/content/mimiciii/1.4/. 10) SIIM-ACR is available at https://www.kaggle.com/c/siim-acr-pneumothorax-segmentation. 11) RSNA is available at https://www.kaggle.com/c/rsna-pneumonia-detection-challenge. 12) NIH ChestX-ray is available at https://nihcc.app.box.com/v/ChestXray-NIHCC. 13) Shenzhen Tuberculosis is available at: https://www.kaggle.com/raddar/tuberculosis-chest-xrays-shenzhen.

## Code availability

The code that supports the findings of this study is available from the corresponding authors upon reasonable request.
