# Supplementary material for: A medical multimodal large language model for future pandemics
Source: NPJ Digit Med. 2023 Dec 2;6:226. doi: 10.1038/s41746-023-00952-2 (PMC10693607; doi:10.1038/s41746-023-00952-2)
Supplement: Supplementary file 1 — Reporting Summary [file 41746_2023_952_MOESM1_ESM.pdf]

nature portfolio

Corresponding author(s): David A. Clifton

Last updated by author(s): Sep 22, 2023

Reporting Summary

Nature Portfolio wishes to improve the reproducibility of the work that we publish. This form provides structure for consistency and transparency in reporting. For further information on Nature Portfolio policies, see our Editorial Policies and the Editorial Policy Checklist.

Statistics

For all statistical analyses, confirm that the following items are present in the figure legend, table legend, main text, or Methods section.

n/a	Confirmed	
☐	☒	The exact sample size (*n*) for each experimental group/condition, given as a discrete number and unit of measurement
☐	☒	A statement on whether measurements were taken from distinct samples or whether the same sample was measured repeatedly
☒	☐	The statistical test(s) used AND whether they are one- or two-sided<br>*Only common tests should be described solely by name; describe more complex techniques in the Methods section.*
☒	☐	A description of all covariates tested
☒	☐	A description of any assumptions or corrections, such as tests of normality and adjustment for multiple comparisons
☐	☒	A full description of the statistical parameters including central tendency (e.g. means) or other basic estimates (e.g. regression coefficient) AND variation (e.g. standard deviation) or associated estimates of uncertainty (e.g. confidence intervals)
☐	☒	For null hypothesis testing, the test statistic (e.g. *F*, *t*, *r*) with confidence intervals, effect sizes, degrees of freedom and *P* value noted<br>*Give P values as exact values whenever suitable.*
☒	☐	For Bayesian analysis, information on the choice of priors and Markov chain Monte Carlo settings
☒	☐	For hierarchical and complex designs, identification of the appropriate level for tests and full reporting of outcomes
☒	☐	Estimates of effect sizes (e.g. Cohen's *d*, Pearson's *r*), indicating how they were calculated

Our web collection on statistics for biologists contains articles on many of the points above.

Software and code

Policy information about availability of computer code

Data collection	No special software and code was used to collect the data.
Data analysis	Python, Pytorch

For manuscripts utilizing custom algorithms or software that are central to the research but not yet described in published literature, software must be made available to editors and reviewers. We strongly encourage code deposition in a community repository (e.g. GitHub). See the Nature Portfolio guidelines for submitting code & software for further information.

Data

Policy information about availability of data

All manuscripts must include a data availability statement. This statement should provide the following information, where applicable:

- Accession codes, unique identifiers, or web links for publicly available datasets
- A description of any restrictions on data availability
- For clinical datasets or third party data, please ensure that the statement adheres to our policy

The data supporting the findings of this study are available within the paper. We have provided a full data availability statement in the manuscript (Please see the Data Availability section). We also have cited any publicly available data on which the conclusions of the paper rely.

Research involving human participants, their data, or biological material

Policy information about studies with human participants or human data. See also policy information about sex, gender (identity/presentation), and sexual orientation and race, ethnicity and racism.

Reporting on sex and gender	N/A. We do not recruit any human research participants for this study. All protected health information (e.g., patient name, sex, gender, and date of birth) is de-identified for all datasets used in our experiments.
Reporting on race, ethnicity, or other socially relevant groupings	N/A. We do not recruit any human research participants for this study. All individual information (e.g., race, ethnicity, or other socially relevant groupings) is not included for all datasets used in our experiments.
Population characteristics	N/A. We do not recruit any human research participants for this study. Deletion of protected health information (PHI) from structured data sources (e.g., database fields that provide age, genotypic information, past and current diagnosis and treatment categories) is performed.
Recruitment	N/A. We do not recruit any human research participants for this study.
Ethics oversight	N/A. We do not recruit any human research participants for this study. All protected personal/health information in our all datasets is de-identified, which is performed in compliance with Health Insurance Portability and Accountability Act (HIPAA) standards in order to facilitate public access to the datasets.

Note that full information on the approval of the study protocol must also be provided in the manuscript.

Field-specific reporting

Please select the one below that is the best fit for your research. If you are not sure, read the appropriate sections before making your selection.

☒ Life sciences ☐ Behavioural & social sciences ☐ Ecological, evolutionary & environmental sciences

For a reference copy of the document with all sections, see nature.com/documents/nr-reporting-summary-flat.pdf

Life sciences study design

All studies must disclose on these points even when the disclosure is negative.

Sample size	To pre-process the data, we randomly partition the dataset into the training, validation, and test sets with a ratio of 8:1:1, respectively.
Data exclusions	No data are excluded from the analyses.
Replication	We conduct five experimental runs with different random seeds and report the mean and standard deviation of the results in our study. In particular, the statistical significance tests indicate that $p < 0.01$. All attempts at replication are successful.
Randomization	We randomly partition the dataset into the training, validation, and test sets with a ratio of 8:1:1, respectively.
Blinding	Blinding is not relevant to the study. All the data collection and analysis (including training and evaluation) are conducted by the computer program automatically.

Reporting for specific materials, systems and methods

We require information from authors about some types of materials, experimental systems and methods used in many studies. Here, indicate whether each material, system or method listed is relevant to your study. If you are not sure if a list item applies to your research, read the appropriate section before selecting a response.

Materials & experimental systems

n/a	Involved in the study	
☒	☐	Antibodies
☒	☐	Eukaryotic cell lines
☒	☐	Palaeontology and archaeology
☒	☐	Animals and other organisms
☒	☐	Clinical data
☒	☐	Dual use research of concern
☒	☐	Plants

Methods

n/a	Involved in the study	
☒	☐	ChIP-seq
☒	☐	Flow cytometry
☒	☐	MRI-based neuroimaging
